# The level of Tim-3^+^CD8^+^ T cells can serve as a potential marker for evaluating the severity of acute graft-versus-host disease after haplo-PBSCT

**DOI:** 10.1590/1414-431X2023e12997

**Published:** 2023-12-18

**Authors:** Nannan Pang, Mingkai Yu, Jianli Xu, Hailong Yuan, Gang Chen, Dong Wang, Chunxia Han, Weiguo Wang, Jianbing Ding, Ming Jiang

**Affiliations:** 1 Department of Pathology, the First Affiliated Hospital of Shihezi University, Shihezi, Xinjiang, China; 2 School of Public Health, Xinjiang Medical University, Urumqi, China; 3 CAS Key Lab of Bio-Medical Diagnostics, Suzhou Institute of Biomedical Engineering and Technology, Chinese Academy of Sciences, Suzhou, China; 4 Center of Hematology, the First Affiliated Hospital of Xinjiang Medical University, Xinjiang Uygur Autonomous Region Research Institute of Hematology, Urumqi, China; 5 Department of Urology, Suzhou Hospital, Affiliated Hospital of Medical School, Nanjing University, Suzhou, China

**Keywords:** haplo-PBSCT, Acute graft-versus-host disease, Tim-3, Galectin-9, Granzyme B

## Abstract

Early and accurate diagnosis of acute graft-*versus*-host disease (aGVHD) after allogeneic hematopoietic stem cell transplantation is crucial for the prognosis of patients. This study identified a potential biomarker for the severity of aGVHD after human leukocyte antigen (HLA)-haploidentical peripheral blood hematopoietic stem cell transplantation (haplo-PBSCT). We included 20 healthy subjects and 57 patients who underwent haplo-PBSCT. Of these patients, 22 developed aGVHD after haplo-PBSCT. The results showed that patients with aGVHD had significantly increased levels of Tim-3^+^/Perforin^+^/Granzyme B^+^CD8^+^ T cells, but significantly decreased Galectin-9. The differences in Galectin-9 and Tim-3^+^/Granzyme B^+^CD8^+^ T cells between grade I-II aGVHD and III-IV aGVHD were also significant. *In vitro*, the apoptosis of CD8^+^ T cells from aGVHD patients was significantly increased after Tim-3/Galectin-9 pathway activation, which decreased Granzyme B secretion. As revealed by univariate analysis, the level of Tim-3^+^CD8^+^ T cells was a risk factor for severe aGVHD. ROC analysis demonstrated that high levels of Tim-3^+^CD8^+^ T cells had a significant diagnostic value for severe aGVHD, with an area under the curve of 0.854 and cut-off value of 14.155%. In conclusion, the binding of Tim-3 with exogenous Galectin-9 can promote apoptosis of CD8^+^ T cells and affect the secretion of Granzyme B. Tim-3^+^CD8^+^ T cells have the potential to serve as immunological markers for assessing the severity of aGVHD after haplo-PBSCT and identifying patients at a higher risk for severe aGVHD.

## Introduction

Allogeneic hematopoietic stem cell transplantation (allo-HSCT) can effectively treat hematopoietic malignancies, especially leukemia (1,2). For patients without conventional donors, an alternative method is haploidentical hematopoietic stem cell transplantation (haplo-HSCT) (3). Acute graft-versus-host disease (aGVHD) and infection are key factors in transplant-related death (TRM) after haplo-HSCT and allo-HSCT efficacy (3,4). The diagnosis of aGVHD typically involves invasive procedures such as liver or intestine biopsy, in addition to clinical manifestations. Nevertheless, accurately diagnosing aGVHD remains a challenge (5). In some cases, patients are already in critical condition once diagnosed, leading to delayed and ineffective treatment (6). Consequently, an objective and timely indicator for early diagnosis of aGVHD and evaluation of aGVHD severity is urgently needed.

aGVHD is an immune-mediated disease that results from the dysfunction of host T cells. Both CD4^+^ T and CD8^+^ T cells are the primary effector cells of aGVHD and are importantly implicated in aGVHD pathogenesis (7,8). Our previous studies showed that CD4^+^ T cell subsets participated in aGVHD (9,10). Th17 cells increased, but Treg cells and TGF-β decreased. Moreover, there was a Th17/Treg functional imbalance in aGVHD patients (9,10). Different from CD4-dependent GVHD, Granzyme/Perforin of CD8^+^ T cells is a crucial molecule resulting in target cell lysis (11,12). Blazar et al. (13) found that CD8^+^ T cells can induce fatal aGVHD in HLA mismatched recipients through the Granzyme/Perforin pathway. Recently, studies have reported that the percentage of Tim-3^+^CD4^+^ and Tim-3^+^CD8^+^ T cells are significantly increased in patients with severe intestinal aGVHD after HLA-matched transplantation, and Tim-3 can be used as a new indicator to predict aGVHD (14,15).

Tim-3 (T cell immunoglobulin and mucin-domain-containing molecule 3) is an immune regulator (16,17). Tim-3 ligands, which include Galectin-9, Psdter, High Mobility Group Box 1 (HMGB1), and Carcinoembryonic antigen-associated cell adhesion molecules (Ceacam-1), exhibit distinct effects upon binding to different ligands on immune cells (18). The binding of Tim-3 to Galectin-9 on activated T cells has been demonstrated to induce apoptosis of T cells and/or inhibit T cell effector functions (19,20). One study found that Tim-3 and Galectin-9 proteins were increased in allografts with acute rejection starting from day 3 and day 10 after lung transplantation, respectively. However, mRNA expression of Galectin-9 did not consistently match its protein levels (21). Shimmura-Tomita et al. (22) reported a significant reduction in the survival time of transplanted cornea and increased infiltration of Tim-3^+^CD8^+^ T cells in the corneal allografts after inhibiting the Tim-3/Galectin-9 pathway. Interestingly, the eye is an immune-privileged organ, and in normal mouse eyes and eyes bearing surviving allografts, Galectin-9 is constitutively expressed on the corneal epithelium, endothelium, and iris-ciliary body. Furthermore, Tim-3 was found to be expressed on CD8 T cells infiltrating the allografts at 4 weeks after transplantation (22). They also found that the expression of Galectin-9 on corneal endothelial cells could protect corneal cells from damage caused by allo-reactive T cells *in vitro* (22). These findings suggest that the expression and rejection reaction of Tim-3/Galectin-9 may vary depending on different study methods, transplanted organs, and transplantation time points. However, the correlation between Tim-3/Galectin-9 and clinical characteristics of aGVHD after HLA-haploidentical peripheral blood hematopoietic stem cell transplantation (haplo-PBSCT) remains unclear. In this context, the objective of this study was to assess the proportion of Tim-3^+^CD8^+^ T cells and the levels of Granzyme B, Perforin, and Galectin-9 after haplo-PBSCT. The potential immune markers for aGVHD evaluation after haplo-PBSCT were also explored.

## Material and Methods

### Study participants

Patients (n=57) newly diagnosed with hematological malignancies from January 2018 to August 2022, who underwent haplo-PBSCT at the Hematologic Disease Center, were included. They all had successful engraftment. The donor cell chimerism rate was greater than 95%, and the patients achieved hematopoietic reconstruction after haplo-PBSCT. The diagnosis and severity of aGVHD followed the Seattle diagnostic criteria (23) and the Chinese Consensus of Allogeneic Hematopoietic Stem Cell Transplantation for Hematological Disease (III) (24). Of these 57 patients, 35 patients did not suffer from aGVHD (non-aGVHD group), and 22 patients suffered from aGVHD (aGVHD group) after haplo-PBSCT, including 16 cases of grade I-II aGVHD and 6 cases of grade III-IV aGVHD (Table 1).

**Table 1:** General information of study participants.

Personal information	HC (n=20)	Patients (n=57)
Age (years) (mean±SD)	35.05±11.92	29.15±11.59
Gender		
Male, n (%)	11 (55.00%)	36 (63.20%)
Female, n (%)	9 (45.00%)	21 (36.80%)
Types of disease, n (%)		
AML		27 (47.37%)
ALL		16 (28.07%)
AA		6 (10.53%)
CML		1 (1.75%)
EL		1 (1.75%)
HPS		2 (3.51%)
MDS		1 (1.75%)
NHL		1 (1.75%)
CMML		1 (1.75%)
MPS		1 (1.75%)
Median infused values (mean±SD)		
aGVHD Grade I-II		
CD34^+^, 10^6^/kg		8.25±5.09
MNC, 10^8^/kg		12.53±4.06
aGVHD Grade III-IV		
CD34^+^, 10^6^/kg		7.89±2.12
MNC, 10^8^/kg		13.50±2.59
Non-aGVHD		
CD34^+^, 10^6^/kg		7.13±2.11
MNC, 10^8^/kg		14.39±3.30
Hematopoietic reconstruction (median, range)		
Neutrophil counts ≥0.5×10^9^/L		14 days (9-21 d)
Platelet counts ≥20×10^9^/L		14 days (7-39 d)
Non-aGVHD, n (%)		35 (61.40%)
aGVHD, n (%)		22 (38.60%)
Grade I-II		16 (72.73%)
Grade III-IV		6 (27.27%)
aGVHD occurrence time (median, range)		
Grade I-II		33 days (11-59 d)
Grade III-IV		38 days (18-57 d)

aGVHD: acute graft-versus-host disease; HC: healthy control; AML: acute myeloid leukemia; ALL: acute lymphoid leukemia; AA: aplastic anemia; CML: chronic myeloid leukemia; EL: eosinophilic leukemia; HPS: hemophagocytic syndrome; MDS: myelodysplastic syndromes; NHL: non-Hodgkin's lymphoma; CMML: chronic myelomonocytic leukemia; MPS: mucopolysaccharidosis; MNC: mononuclear cells; d: days.

Blood samples were collected from the patients on day 30 after transplantation. If aGVHD occurred before day 30 after transplantation, peripheral blood was collected immediately. Meanwhile, peripheral blood samples were collected from 20 healthy controls (HC group). The Ethics Committee of the First Affiliated Hospital of Xinjiang Medical University in China approved this study (approval No. 20101203). All subjects signed an informed consent form.

### Myeloablative conditioning regimen, PBSCT collection, and infusion

The myeloablative conditioning regimen included Ara-C (days -9 and -8, 2-4 g/m^2^) + busulfan (days -7 and -5, 3.2 mg/kg per day) + cyclophosphamide (days -3 and -2, 1.8 mg/m^2^ per day) + anti-thymocyte immunoglobulin (ATG) (2.5 mg/kg per day; Genzyme, USA; intravenous infusion, days -4 ∼ -1). Peripheral blood mononuclear cells (PBMCs) were mobilized with 7-10 µg/kg per day of granulocyte-colony stimulating factor. The infusion dose was (14.3±5.32) × 10^8^/kg for mononuclear cells (MNC) and (9.58±4.62) × 10^6^/kg for CD34^+^ cells.

### Prevention and treatment of aGVHD

GVHD prophylaxis included cyclosporine A (day -5 to +30, 2.5 mg/kg, *iv*; maintenance concentration 200-300 ng/mL; 4-5 mg/kg for oral administration on day +30 to +100, with trough concentration between 100-300 ng/mL, and discontinuation on day 100), anti-CD25 mAb (day +1 and day +2, 12 mg/m^2^, *iv*; Novartis Pharma Schweiz AG, Switzerland), mycophenolate mofetil (day -1 to +100, 0.5 g orally BID), short-term methotrexate (day +1, 15 mg/m^2^, *iv*; day +3 to +6, 10 mg/m^2^), and short-range glucocorticoid (5 mg dexamethasone, *iv*, on day +1 to +15, with gradually decreasing dose and discontinuation on day +30). Among the 22 patients with aGVHD, 19 patients had obvious recovery after treatment and 3 patients developed chronic GVHD on days 128, 150, and 154 after transplantation.

### Flow cytometry detection

Peripheral blood samples (200 μL each) from both the patients and the HCs were incubated with fluorescently labeled antibodies of CD8-FITC (Cat# 555634), Granzyme B-Alexa Fluor^®^647 (Cat# 561999), Perforin-PerCP-Cy^TM^5.5 (Cat# 563762), and Tim-3-PE (Cat# 563422). IgG-PE (Cat# 560951) served as the negative isotype control. First, the cells were labeled with CD8-FITC and Tim-3-PE antibodies. Next, the cells were treated with Fix/Per (Fix/Per buffer) and then stained for Granzyme B and Perforin. Finally, cells were detected on the Calibur flow cytometer (BD Biosciences, USA) and processed with Kaluza 2.1 software (Beckman-Coulter, USA). For the gating strategy, the lymphocytes were first gated by SSC and FSC, and then CD8^+^ T cells were further gated. Tim-3, Granzyme B, and Perforin levels in CD8^+^ T cells were also evaluated.

### Flow cytometry analysis of apoptotic cells

Cells were stained using Annexin V-FITC and PI for 20 min in the dark according to the manufacturer’s instructions (FITC Annexin V Apoptosis Detection Kit). Cell populations were separated as follows: viable cells: Annexin V-/PI-; early apoptosis: Annexin V+/PI-; late apoptosis: Annexin V+/PI+; dead cells: Annexin: V-/PI+. BD Biosciences provided the antibodies and reagents.

### ELISA

Serum samples from patients after haplo-PBSCT and HCs were used. The serum levels of Galectin-9 were determined using an ELISA kit from Invitrogen (Cat# EH206RBX10; USA), while the levels of Perforin and Granzyme B in the supernatants were measured using ELISA kits from Abcam (Perforin: Cat# ab46068, Granzyme B: Cat# ab46142; USA). The absorbance (OD 450 value) was measured with a microplate reader (Thermo Fisher, USA). The concentrations of Galectin-9, Perforin, and Granzyme B were calculated following the standard curve. The detection ranges of Galectin-9, Granzyme B, and Perforin were 36.86-9000 pg/mL, 31.25-1000 pg/mL, and 62.5-2000 pg/mL, respectively.

### Isolation of CD8^+^ T cells from aGVHD and non-aGVHD patients and cell treatment

PBMCs were isolated from peripheral blood by Ficoll (Cat# P8900, Solarbio, China). Briefly, peripheral blood and PBS buffer were diluted at a ratio of 1:1 and fully mixed. Then, an equal volume of lymphocyte separation solution was added and the mixture was centrifuged at 500 *g* for 20 min at 4°C. The white membrane cells were carefully extracted and washed with PBS. Subsequently, the cells were centrifuged at 300 *g* for 10 min at 4°C. CD8^+^ T cells from PBMC of aGVHD patients and non-aGVHD patients were sorted using the CD8^+^ T cell isolation kit (Cat# 130-096-495; Miltenyi Biotec, Germany). The purity of the isolated cells was confirmed to be over 95% by flow cytometry. After incubation with CD3 (clone OKT3; Cat# 16-0037-85) and CD28 (CD28.2, 0.5 μg/mL; Cat# 16-0289-85) (eBioscience, USA) for 48 h *in vitro*, the sorted CD8^+^ T cells were further incubated with Galectin-9 recombinant protein (Cat# LG9-H5244, 5 μg/mL; ACRO Biosystems, USA) or PBS at 37°C for 24 h. After that, both the cells and culture supernatants were collected. Cell apoptosis was analyzed with flow cytometry, while cytokine levels in the supernatants were measured with ELISA.

### Statistical analysis

Data were processed with SPSS 26.0 (IBM, USA). *T*-test or analysis of variance was used to test the difference of measurement data, which are reported as means±SD. The chi-squared test was used to compare the difference of count data, and the Spearman's correlation test was used to analyze the data correlation. Meanwhile, univariate analysis was used to assess the key risk factors affecting the severity of aGVHD. Finally, the receiver operating characteristic (ROC) curve was used to evaluate the diagnostic performance. All statistical tests were performed with a significance level of P<0.05.

## Results

### Patients and specimens

A total of 77 participants, consisting of 57 patients and 20 HCs, were included in the study (Table 1). There were no significant differences in age and gender between the patients and HCs (P>0.05).

### Tim-3^+^CD8^+^ T cell proportion was increased while Galectin-9 levels were decreased in aGVHD patients

**Figure 1:**
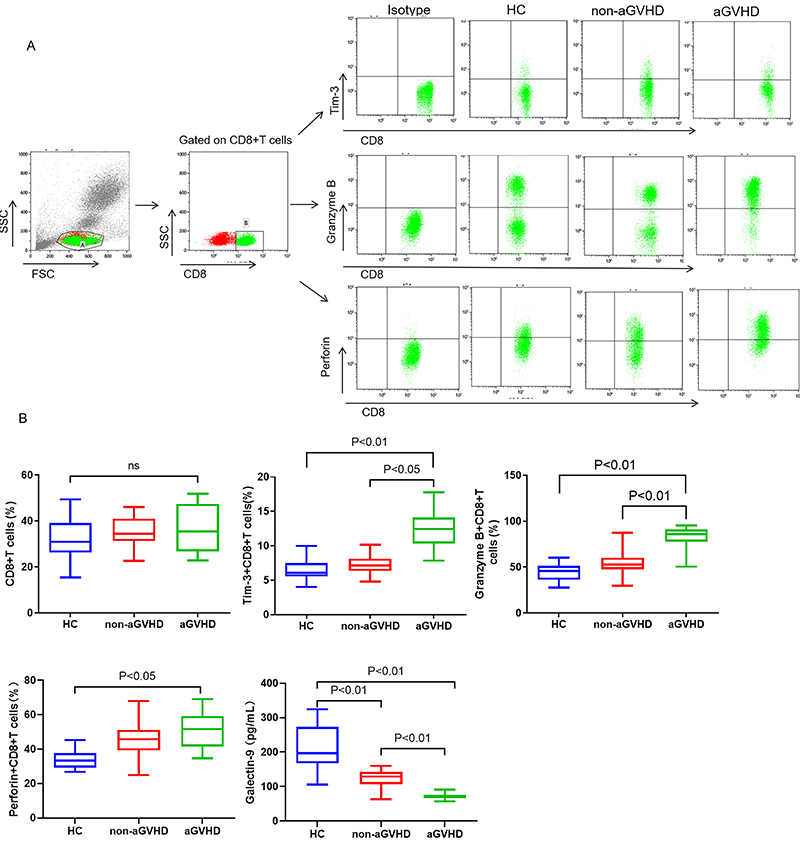
Level of Tim-3^+^CD8^+^ T, Granzyme-B^+^CD8^+^ T, Perforin^+^CD8^+^ T cells, and Galectin-9 in patients with or without acute graft-versus-host disease (aGVHD) after transplantation. Flow cytometry measured the proportions of CD8^+^ T cell subsets in peripheral blood of healthy control (HC), non-aGVHD, and aGVHD groups after transplantation. Cells were gated on CD8^+^ T cells (**A**). Numbers within each quadrant represent the percentages of cells within each dot plot. **B**, Comparisons of the percentages of CD8^+^ T cells, Tim-3^+^CD8^+^ T cells, Granzyme-B^+^CD8^+^ T cells, Perforin^+^CD8^+^ T cells, and Galectin-9 in peripheral blood of HC, non-aGVHD, and aGVHD groups. Data are reported as median and interquartile range (Kruskal-Wallis test). ns: not significant.

Flow cytometry was used to measure the percentage of Tim-3^+^/Granzyme B^+^/Perforin^+^CD8^+^ T cells in peripheral blood. The results showed that their percentages increased in patients after haplo-PBSCT. No significant difference in CD8^+^ T cells and Perforin^+^CD8^+^ T cells was found between non-aGVHD and aGVHD group (Figure 1A and B). However, the aGVHD group had significantly greater percentages of Tim-3^+^CD8^+^ T cells and Granzyme B^+^CD8^+^ T cells than the non-aGVHD group (P<0.05) (Figure 1A and B). ELISA results showed that serum Galectin-9 levels were significantly decreased in the aGVHD group (P<0.01) (Figure 1B).

### Analysis of Galectin-9 levels and Tim-3^+^/Granzyme B^+^CD8^+^ T cells in grade I-II and III-IV aGVHD

**Figure 2:**
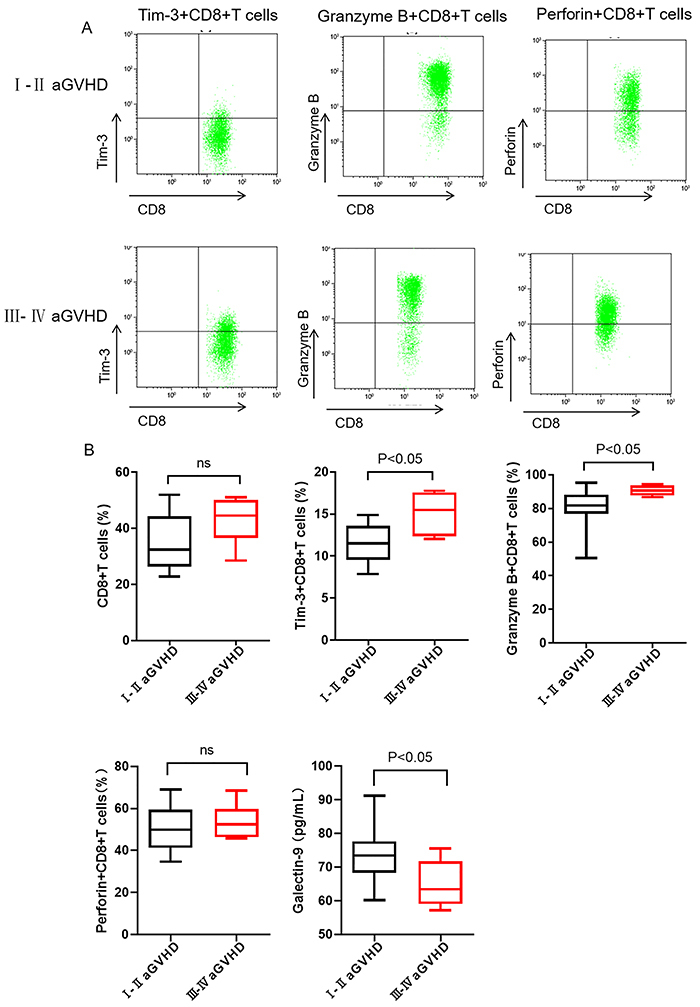
**A**, Flow cytometry dot plots of Tim-3^+^CD8^+^ T cells, Granzyme-B^+^CD8^+^ T cells, and Perforin^+^CD8^+^ T cells in patients with various grades of acute graft-versus-host disease (aGVHD) after transplantation. **B**, Percentages of CD8^+^ T cells, Tim-3^+^CD8^+^ T cells, Granzyme-B^+^CD8^+^ T cells, Perforin^+^CD8^+^ T cells, and Galectin-9 level in aGVHD patients (grades I-II and III-IV). Data are reported as median and interquartile range (Mann Whitney U-test). ns: not significant.

**Figure 3:**
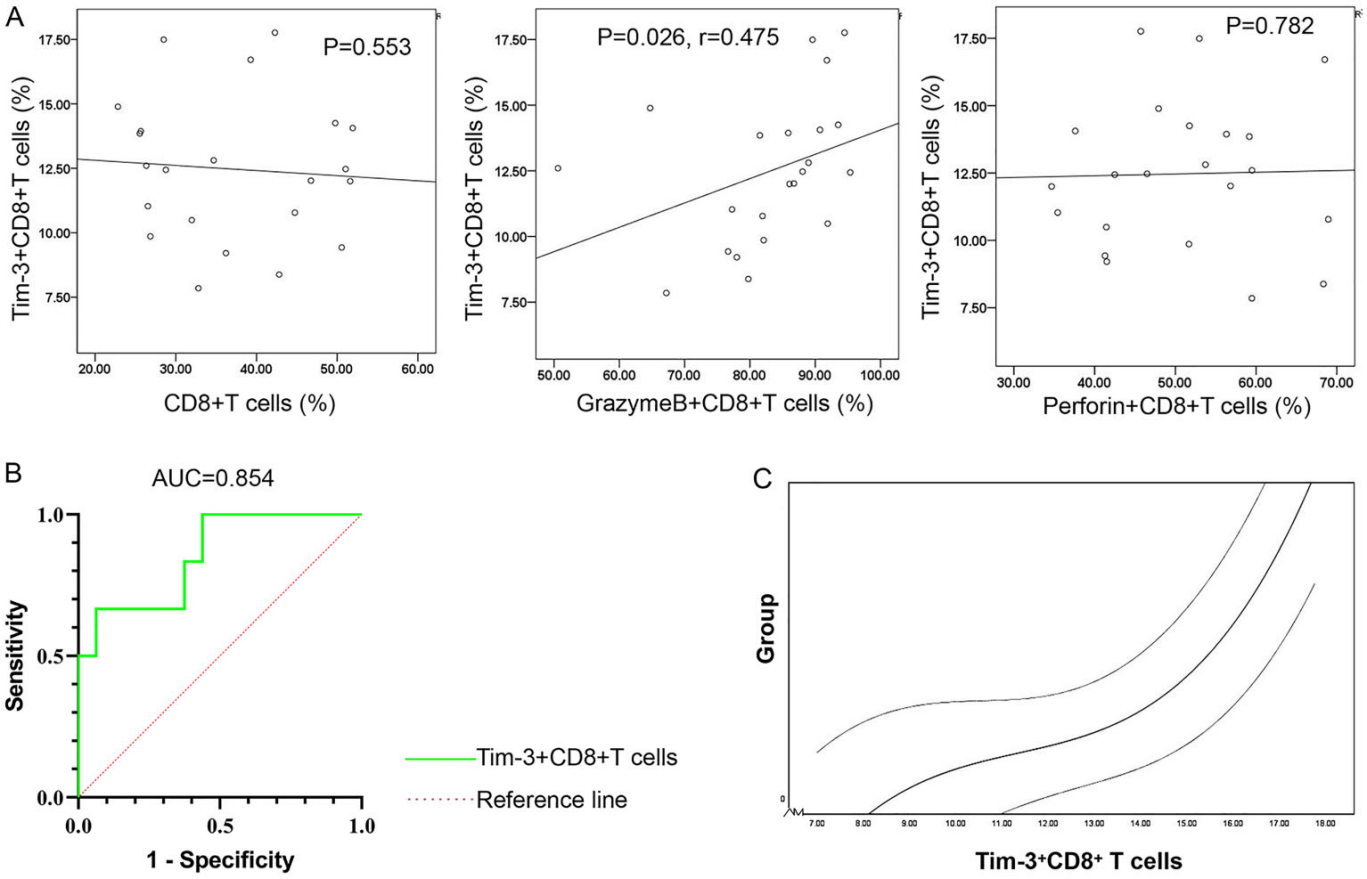
**A**, Spearman correlation analysis of Tim-3^+^CD8^+^ T cells in acute graft-versus-host disease (aGVHD) patients. **B**, ROC analysis for diagnostic value of Tim-3^+^CD8^+^ T cells for aGVHD severity. **C**, The relationship of Tim-3^+^CD8^+^ T cells with aGVHD severity was analyzed with the smooth curve fitting. Light gray lines represent the upper and lower 95% confidence intervals.

The percentage of Tim-3^+^CD8^+^ T cells in the III-IV aGVHD group was significantly higher than that in the I-II aGVHD group (Figure 2A), whereas the Galectin-9 level in the III-IV aGVHD group was significantly lower than that in I-II aGVHD group (P<0.05) (Figure 2B). Additionally, the percentages of CD8^+^ T cells and Perforin^+^CD8^+^ T cells were not significantly different between I-II and III-IV aGVHD groups (Figure 2). Furthermore, the correlation of CD8^+^ T cells, Tim-3^+^CD8^+^ T cells, Granzyme B^+^CD8^+^ T cells, and Perforin^+^CD8^+^ T cells was evaluated. As shown in Figure 3A, there was a positive correlation between the Granzyme B^+^CD8^+^ T cells and Tim-3^+^CD8^+^ T cells in aGVHD patients (r=0.475, P<0.05). Thus, Tim-3^+^CD8^+^ T cells, Granzyme B, and Galectin-9 may be related to aGVHD severity.

### Analysis of risk factors for aGVHD severity

The above results showed significant differences in the percentages of Granzyme B^+^CD8^+^ T cells and Tim-3^+^CD8^+^ T cells, as well as Galectin-9 levels, between the I-II aGVHD and III-IV aGVHD groups. In the univariate analysis, the continuous variables age, CD8^+^ T cells, Tim-3^+^/Granzyme B^+^/Perforin^+^CD8^+^ T cells, and Galectin-9 and categorical variables gender and disease risk were used as independent variables. Meanwhile, the I-II and III-IV aGVHD served as the outcome-dependent variable. We found that high levels of Tim-3^+^CD8^+^ T cells (OR=2.059, 95%CI: 1.081-3.922, P=0.028) was the risk factor for severe aGVHD (Table 2).

**Table 2:** Risk factors for severe acute graft-versus-host disease (aGVHD) by univariate analysis.

Factors	Statistics n (%)	OR (95%CI)	P-value
Age (years)	22	1.001 (0.933, 1.074)	0.977
Gender			
Male, n (%)	14 (63.64%)		
Female, n (%)	8 (36.36%)	0.833 (0.115, 6.013)	0.857
Disease risk*			
High-risk, n (%)	14 (63.63%)		
Standard risk, n (%)	6 (27.27%)	0.073 (0.165, 10.743)	0.787
CD8^+^ T cells (%)	22	1.091 (0.980, 1.214)	0.112
TIM-3^+^CD8^+^ T cells (%)	22	2.059 (1.081, 3.922)	**0.028**
Granzyme-B^+^CD8^+^ T cells (%)	22	1.284 (1.000, 1.647)	0.050
Perforin^+^CD8^+^ T cells (%)	22	1.037 (0.944, 1.138)	0.449
Galectin-9 (pg/mL)	22	0.816 (0.671, 0.992)	**0.041**

Disease risk was missing for 2 patients. P-values in bold indicate statistical significance.

### Predictive value of Tim-3^+^CD8^+^ T cells for aGVHD severity

The ROC curve was used to indicate the predictive value of Tim-3^+^CD8^+^ T cells for aGVHD severity. The results showed that Tim-3^+^CD8^+^ T cells had a high predictive value for aGVHD severity. The area under the curve (AUC) value was 0.854, the sensitivity was 0.667, and the specificity was 0.937. The cut-off value for diagnosing severe aGVHD was 14.155% (95%CI: 0.675-1.000, Youden index=0.604, P<0.05) (Figure 3B). The changing trend of the fitted curve of Tim-3^+^CD8^+^ T cells was consistent with the severity diagnosis of aGVHD as determined by the ROC analysis (Figure 3C). These findings provided further evidence of the potential of Tim-3^+^CD8^+^ T cells as an immune marker for assessing aGVHD severity.

### Galectin-9 recombinant protein promoted the apoptosis of CD8^+^ T cells by activating Tim-3/Galectin-9 pathway *in vitro*

**Figure 4:**
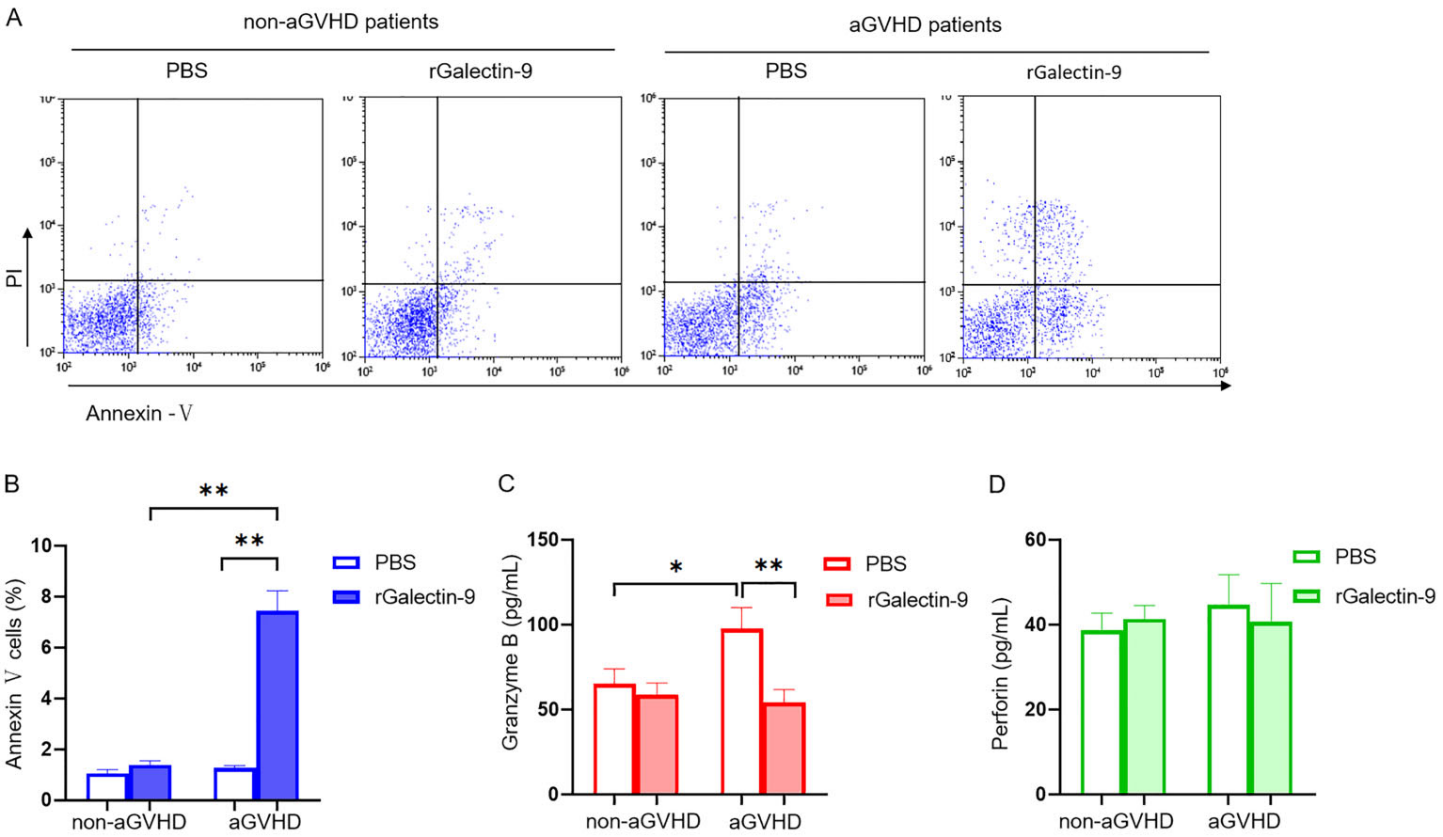
Activation of the Tim-3/Galectin-9 pathway with rGalectin-9 induces apoptosis of CD8^+^ T cells *in vitro*. **A**, Isolated CD8^+^ T cells were cultured with Galectin-9 recombinant protein (rGalactin-9) for 24 h. Flow cytometry determined cell apoptosis. The total percentage of apoptosis is equal to the percentage of early apoptosis (Annexin V+/PI-) plus the percentage of late apoptosis (Annexin V+/PI+). **B**, Quantitative flow cytometry results. Levels of Granzyme B (**C**) and Perforin (**D**) in the supernatants with or without rGalectin-9 were tested by ELISA. Data are reported as mean and SD. *P<0.05; **P<0.01, one-way ANOVA was used for comparing apoptosis, Granzyme-B, and Perforin between the two groups.

We sorted out CD8^+^ T cells from grade I-II aGVHD patients and non-aGVHD patients. CD8^+^ T cells were first intervened for 48 h with CD28 and CD3, and then with Galectin-9 recombinant protein for 24 h *in vitro*. Flow cytometry showed that, compared to that of non-aGVHD patients and the PBS group, the apoptosis of CD8^+^ T cells from aGVHD patients significantly increased in the Galectin-9 group (Figure 4A and B) (P<0.05). Perforin and Granzyme B levels in the culture supernatant were detected by ELISA. The Granzyme B level of aGVHD patients was increased in the PBS group and it was significantly decreased after activating the pathway by Galectin-9 recombinant protein (P<0.05) (Figure 4C). Nevertheless, the level of Perforin in the rGalectin-9 group was not significantly different from that in the PBS group (P>0.05) (Figure 4D). Therefore, apoptosis and cytokines secreted by CD8^+^ T cells were affected by the Tim-3/Galectin-9 pathway in aGVHD patients.

## Discussion

Clinically significant aGVHD affects a substantial 40 to 50% of allo-HSCT recipients (25). The proliferation, activation, and differentiation of T cells has a critical role in GVHD development (26). CD8-induced GVHD primarily depends on the cytolytic machinery. Perforin and Granzymes of CD8^+^ T cells can promote the production of proinflammatory cytokines, which continually fuel GVHD (26). Several studies have confirmed that abnormalities of some serum cytokines are associated with aGVHD (27-29). However, because of the complex pathogenesis and multifactorial nature of aGVHD, there are currently no specific immunological markers available for the prediction and risk assessment of aGVHD. Thus, there is an urgent need to identify non-invasive immune biomarkers for diagnosing aGVHD. Here, our study revealed decreased levels of Galectin-9 but increased levels of Granzyme B and a higher proportion of Tim-3^+^CD8^+^ T cells in patients with severe aGVHD. Furthermore, ROC analysis demonstrated that Tim-3^+^CD8^+^ T cells have high diagnostic accuracy for determining the severity of aGVHD. Measurement of Tim-3^+^CD8^+^ T cells may facilitate the early identification of patients at high risk for severe aGVHD.

Tim-3, one of the important members of the Tim family, plays a key role in regulating the differentiation and proliferation of CD4^+^ T and CD8^+^ T cells and is involved in the occurrence and development of many diseases such as tumors, infections, and autoimmune diseases (19,30). Splenic and hepatic CD4^+^ T and CD8^+^ T cells, especially hepatic CD8^+^ T cells, have high Tim-3. However, the anti-Tim-3 treatment enhances effector T cell activation in aGVHD, leading to increased IFN-γ expression or cytotoxicity in a mouse aGVHD model (31). Our study showed that the levels of Perforin, Granzyme B, and Tim-3^+^CD8^+^ T cells increased significantly in the peripheral blood of patients with aGVHD after haplo-PBSCT, and Granzyme B correlated positively with the percentage of Tim-3^+^CD8^+^ T cells. These results demonstrated that the overexpression of Tim-3 on CD8^+^ T cells may be related to abnormal activation of CD8^+^ T cells in aGVHD patients. McDonald et al. (32) reported that IL-6, Tim-3, Stimulation-2 (ST2), and sTNFR1 levels in plasma could predict the development of non-relapse mortality and more severe GVHD in patients with gastrointestinal GVHD. We further found that patients with severe aGVHD exhibited a substantial elevation in the levels of Tim-3^+^CD8^+^ T cells and Granzyme B compared to patients with mild aGVHD, and univariate analysis confirmed that the level of Tim-3^+^CD8^+^ T cells was a risk factor for severe aGVHD. Tim-3^+^CD8^+^ T cell level (>14%) had a high diagnostic value for aGVHD severity. It was implied that Tim-3^+^CD8^+^ T may aggravate aGVHD by secreting Granzyme, Perforin, etc. Therefore, Tim-3^+^CD8^+^ T cells have the potential to serve as a novel immune marker for predicting severe aGVHD.

Tim-3 can induce immune tolerance after organ transplantation through its unique function of immune negative regulation (33). Liu et al. (34) found that the Tim-3 molecule exhibited a negative regulatory effect on T cells and suppressed the Tim-3/Galectin-9 pathway, which aggravated the immune rejection reaction in a mouse model. Galectin-9, as a ligand of Tim-3, can down-regulate the immune response after binding to Tim-3 on helper T cells (Th1 and Th17) and CD8^+^ T cells by stimulating apoptosis of mature T cells (35). Recently, a study found that patients without aGVHD had elevated Galectin-9 levels compared to normal control and patients with aGVHD; thus, a high Galectin-9 level represented a potential prognostic biomarker of aGVHD after allo-HSCT (36). We found that Tim-3 was over-expressed on CD8^+^ T cells but Galectin-9 level was significantly decreased in aGVHD patients. *In vitro*, Galectin-9 not only induces apoptosis of CD8^+^ T cells but also inhibits T cell receptor cross-linking proliferation and down-regulates the cytotoxicity of activated CD8^+^ T cells (37). Our study suggested that insufficient expression of Galectin-9 may not effectively suppress overactivated Tim-3^+^CD8^+^ T cells, leading to aGVHD. Therefore, we believe that at 15-45 days post-transplantation, the proportion of Tim-3^+^CD8^+^ T cells in patients should be dynamically monitored weekly. An increase in the proportion of Tim-3^+^CD8^+^ T cells may indicate the potential occurrence of aGVHD, especially when the proportion exceeds 14.55%. These elevated Tim-3^+^CD8^+^ T cells may serve as potential immune markers for diagnosing severe aGVHD.

In a mouse skin transplantation model, exogenous recombinant Galectin-9 could reduce allograft rejection and significantly prolong the survival time of skin grafts (37,38). In addition, Galectin-9 selectively binds to effector T cells expressing Tim-3, leading to their apoptosis, inhibition of T cell activation, and promotion of Treg cell function. As a result, it plays a crucial role in regulating the body's immune response (39). Graft survival after haplo-HSCT is associated with factors such as the use of granulocyte colony-stimulating factor, clearance of memory T cells in the recipient (anti-thymocyte globulin, ATG), and induction of T cell immune tolerance (40). In our study, all patients achieved successful engraftment after transplantation. Therefore, the results demonstrated that the apoptosis of Tim-3^+^ T cells induced by Galectin-9 after transplantation not only had no adverse effect on graft survival but also created a favorable bone marrow microenvironment for long-term graft survival in the recipient. Interestingly, we found that after activating the Tim-3/Galectin-9 pathway *in vitro*, the apoptosis of CD8^+^ T cells was significantly enhanced while the secretion of Granzyme B was reduced. These findings suggested that the combination of Galectin-9 and Tim-3 could effectively suppress the immune response of CD8^+^ T cells after haplo-PBSCT, which could become a new immunological method for the treatment or prevention of aGVHD in the future.

Our study, however, does have certain limitations. Firstly, our study's sample size was relatively small, and future studies should include more cases for further analysis. Secondly, our study did not include the testing of ST2, a biomarker for predicting more severe gastrointestinal GVHD (35). Further investigation is required to explore the relationship between ST2 and Tim-3^+^CD8^+^ T cells.

In conclusion, we demonstrated that Tim-3^+^CD8^+^ T cells and Galectin-9 were significantly involved in aGVHD. The level of peripheral blood Tim-3^+^CD8^+^ T cells was elevated in aGVHD patients, which might serve as an immune diagnostic marker to assess disease severity.
